# New Mechanism of Spiral Wave Initiation in a Reaction-Diffusion-Mechanics System

**DOI:** 10.1371/journal.pone.0027264

**Published:** 2011-11-14

**Authors:** Louis D. Weise, Alexander V. Panfilov

**Affiliations:** 1 Department of Theoretical Biology, Utrecht University, Utrecht, The Netherlands; 2 Department of Physics and Astronomy, Ghent University, Ghent, Belgium; University of Maribor, Slovenia

## Abstract

Spiral wave initiation in the heart muscle is a mechanism for the onset of dangerous cardiac arrhythmias. A standard protocol for spiral wave initiation is the application of a stimulus in the refractory tail of a propagating excitation wave, a region that we call the “classical vulnerable zone.” Previous studies of vulnerability to spiral wave initiation did not take the influence of deformation into account, which has been shown to have a substantial effect on the excitation process of cardiomyocytes via the mechano-electrical feedback phenomenon. In this work we study the effect of deformation on the vulnerability of excitable media in a discrete reaction-diffusion-mechanics (dRDM) model. The dRDM model combines FitzHugh-Nagumo type equations for cardiac excitation with a discrete mechanical description of a finite-elastic isotropic material (Seth material) to model cardiac excitation-contraction coupling and stretch activated depolarizing current. We show that deformation alters the “classical,” and forms a new vulnerable zone at longer coupling intervals. This mechanically caused vulnerable zone results in a new mechanism of spiral wave initiation, where unidirectional conduction block and rotation directions of the consequently initiated spiral waves are opposite compared to the mechanism of spiral wave initiation due to the “classical vulnerable zone.” We show that this new mechanism of spiral wave initiation can naturally occur in situations that involve wave fronts with curvature, and discuss its relation to supernormal excitability of cardiac tissue. The concept of mechanically induced vulnerability may lead to a better understanding about the onset of dangerous heart arrhythmias via mechano-electrical feedback.

## Introduction

Reaction-diffusion (RD) equations describe a wide range of phenomena in biological, physical and chemical systems, such as rotating spiral waves. Examples are spiral waves in the Belousov-Zhabotinsky (BZ) reactions [1], [2] and in the catalyzed oxidation of carbon monoxide on platinum surfaces [3]. Spiral waves lead the morphogenesis of the *Dictyostelium discoideum* amoebae [4], [5] and occur in retinal and cortical nerve tissue [6], where they underpin neurological diseases, such as epilepsy and migraine. One of the most studied systems is the heart, where spiral wave excitation patterns are a main cause for life-threatening cardiac arrhythmias [2], [7].

In many cases the phenomena described by the RD equations are closely coupled with mechanical processes, such as cell motion in Dd-morphogenesis [5] or the swelling of a gel caused by BZ reactions [8], that cannot be described by the RD equations alone. For the heart, the coupling mechanism between the excitation and the deformation processes works in both directions. The primary physiological function of the heart, its rhythmical pumping, is governed by electrical waves of excitation [9]. The contraction of the heart, however, in turn directly affects the excitation process of the cardiomyocytes. This phenomenon ‘mechano-electrical-feedback’ has been studied in cardiac electrophysiology for over a century, and has been shown to have both positive and negative consequences on the heart rhythm [10]. Recently, a modeling approach has been proposed, that can account for basic effects of the coupled electrical and mechanical cardiac activity, the reaction-diffusion mechanics (RDM) modeling framework [11]. The RDM approach combines the RD equations to describe wave propagation with the equations of continuum mechanics to model the deformation of the medium. Using this RDM framework, important phenomena were identified, such as self-organized pacemakers [12] and the drift and breakup of rotating spiral waves [13].

In this paper we show results of a RDM study on an initial step of the onset of cardiac arrhythmias, the initiation of spiral waves. For this research we applied a recently introduced discrete RDM model [14] (dRDM), which couples FitzHugh-Nagumo RD equations for cardiac excitation [15] with a discrete mechanics model describing a finite elastic, isotropic material (Seth material). The dRDM framework describes cardiac excitation-contraction coupling and immediate mechano-electrical-feedback due to a depolarizing stretch activated current.

Vulnerability of an excitable medium is the phenomenon of spiral wave initiation by a local stimulus whose responding wave is unidirectionally blocked by an asymmetric distribution in excitability [16]. We denote the vulnerable zone at the refractory tail of an excitation wave in this paper as the ‘classical vulnerable zone’. We studied vulnerability in the dRDM model with the ‘pinwheel experiment’, a standard stimulation protocol for the initiation of spiral waves. In this protocol a stimulus is applied in the back of a previously initiated excitation wave, with a certain coupling interval between the applied stimuli [17], [18].

Our main finding is that deformation induces a new type of vulnerability in the dRDM system which results in a new mechanism of spiral wave initiation. In this mechanism an unidirectional block occurs in the opposite direction compared to a block caused in the ‘classical vulnerable zone’. This mechanically caused unidirectional block results in pairs of spiral waves rotating in the opposite direction to those resulting from a wave block in the ‘classical vulnerable zone’. We study the mechanism of this phenomenon and its dependence on changes in the stimulation protocol. Furthermore, we provide examples when this new type of spiral wave initiation naturally occurs due to stretch caused by wave fronts with curvature. Finally, we discuss the importance of our findings for the phenomena of supernormal excitability of cardiac tissue and the onset of cardiac arrhythmias.

## Methods

For this study we used the dRDM model [14], which has been shown to enable efficient computations with higher spatial and temporal numerical resolution compared to the continuous RDM description used in [12]. In [14] a detailed description of the dRDM model setup and numerical methods is given. Table 1 provides an overview of all parameters of the dRDM model and their numerical values used in this publication and shows alterations to parameters used in [14].

**Table 1:** Overview of dRDM parameters.

Category	Parameter	Numeric value
Electrophysiology		
	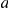	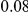 ; ( 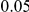 )
	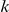	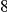
Mechanics		
	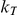	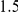
	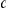	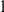 , varied in inhomogeneities; ( 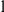 )
	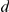	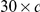
	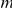	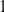
Electromechanical feedback		
	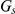	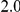 and 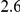 ; ( 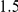 )
	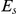	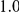
Mesh coupling		
	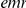	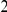
	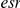	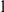
	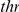	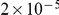
Integration		
	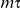	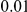
	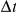	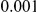
	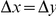	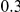

Parameters for the dRDM model used in this paper, and after a semicolon ‘;’ (in parentheses) numerical values are shown that were used in the previous publication [14] if the numerical value changed in the present paper. Parameter 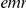 is the ratio of mass points to finite difference points, 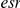 is the update ratio of the RD system to the update of the mesh configuration.

The dRDM model consists of a two-variable FitzHugh-Nagumo type RD model for cardiac excitation [15], coupled with mechanics equations describing a finite-elastic, isotropic material [14]

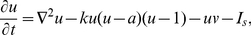
(1)




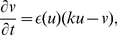
(2)




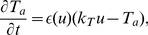
(3)

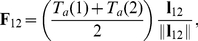
(4)

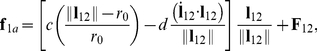
(5)

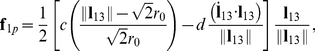
(6)

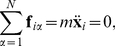
(7)

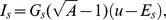
(8)


where 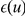 is a step function setting the time scale of the recovery and contraction process: 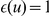 for 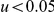, and 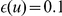 for 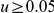. For undeformed tissue, Eqs.(1),(2) with transmembrane potential 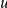 (in dimensionless excitation units [e.u.]) and conductance of slow repolarizing current 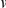 (in dimensionless recovery units [r.u.]), describe non-oscillatory cardiac tissue, which is able to provide stable wave propagation for the parameters 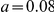 (activation threshold) and 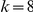 (magnitude of transmembrane current) used in this paper. The medium of the dRDM model is described by a square lattice, where mass points are connected to 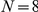 (if not at the boundary) direct neighboring mass points with springs. Each such mass point is connected to its 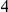 diagonal neighbors with ‘passive springs’ (describing passive elastic properties only), and to its 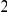 vertical and 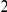 horizontal neighbors with ‘active springs’ (springs that additionally mediate active forces). Eqs.(3),(4) describe the excitation-contraction coupling for two neighboring mass points 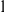 and 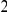 that are connected by an active spring, where 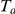 modulates the active contraction force via Eq.(4), and the parameter 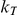 (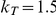 in this paper) controls the amplitude of the contraction twitch. The Eqs.(5),(6) describe the forces 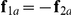 mediated through an active spring to mass points 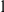 and 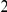, and the forces 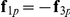 mediated through a passive spring to mass points 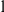 and 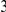, respectively. In Eqs.(5),(6) the spring vectors are given by the mass point's positions as 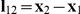 and 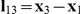, 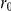 is the resting length of an active spring and 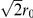 the resting length of a passive spring, 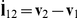 and 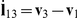 are the time derivatives of the respective spring vectors 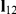 and 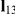, and 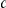 and 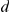 are the stiffness and damping constants (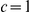 unless stated otherwise, and 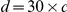). The forces 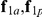 are functions of the mass point's positions 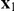, 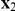, 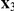, velocities 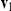, 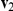, 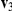 and 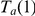, 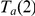 that form the active force distribution through Eqs.(3),(4). Following previous RDM studies to study cardiac electromechanics [11]–[14] elastostatics was assumed in this paper. By solving Eq.(7), with the mass 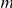 of a mass point set to 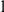, the elastostatic configuration of the lattice for every force distribution defined by the RD state is found. The deformation of the tissue feeds back on the excitation process. Following previous studies [12]–[14], we describe the direct influence of deformation on the excitation state of the medium as depolarizing stretch-activated currents [10], that are activated instantaneously with mechanical dilatation, and describe a linear current-voltage relationship [19], [20]. Therefore, we use Eq.(8) in this paper to model mechano-electrical feedback through stretch activated channels. In Eq.(8) the parameters 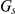 and 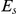 are the maximal conductance and reversal potential of the stretch activated channels (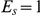 in this paper), the variable 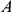 is the normalized surface area (relative to the undeformed reference surface area) of a square, formed by 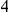 direct neighboring mass points, connected with active springs. Stretch activated current 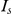 is active only if 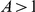 (stretch). The value of 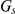 is a main parameter of the effect of 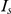. In our simulations we varied 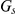 between 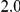 and 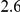, feedback strengths where also pacemaking activity [12], [14] can occur in the medium.

### Numerical Methods

The coupled dRDM model was solved with a hybrid approach, the explicit Euler method to solve the RD system, and the Verlet integration scheme [21] to solve the mechanical model. After each Euler computation of the RD system describing a new force distribution in the medium, the mechanical equations were solved with a Verlet integration time step 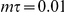 until the sum of forces for each mass point was smaller than the threshold 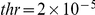 (dimensionless force units [f.u.]). Euler computations were performed on a quadratic deforming grid of up to 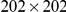 finite difference points using no-flux boundary conditions. For all simulations, an Euler integration time step of 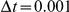 (dimensionless time units [t.u.]) and a space integration step of 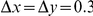 (dimensionless space units [s.u.]) were used. According to the explicit Euler method the diffusion term in Eq.(1) for a electrical mesh point at position 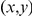 is computed as
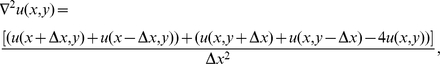



and the excitation state of an electrical mesh point for a next time step 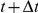 is computed as
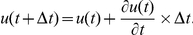



The position of a mass point 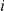 during the iterative solution of Eq.(7) is computed by a Verlet step with
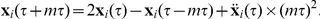



The boundaries of the deformable medium were fixed in space modeling isometric contraction to mimic isovolumic phases in the cardiac cycle, an assumption that has been made also in previous electromechanical studies [12], [14]. The parameter 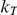 together with the stiffness constant 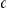 control local deformations of the contraction process. These parameters were chosen to give rise to relative local deformations in the medium of up to 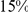 (similar to contracting cardiac cells). The elastic properties of this model can be described by the Seth material constitutive relation [14].

### Pinwheel Experiment

**Figure 1:**
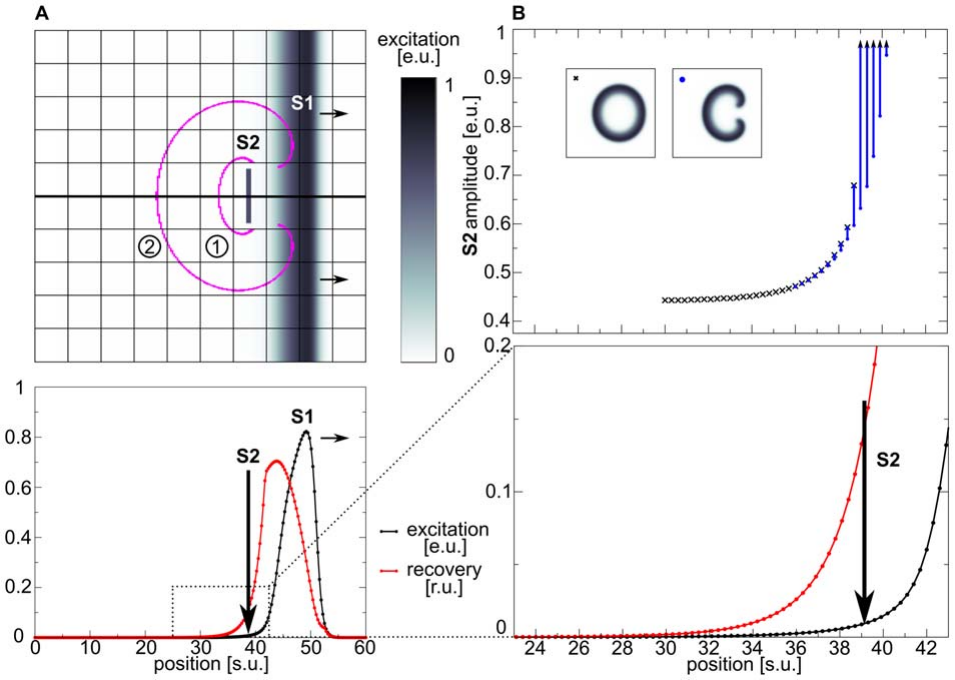
Pinwheel experiment. (**A**) Spiral wave initiation by S2 stimulus in ‘classical vulnerable zone’. Upper panel shows 2D medium. The resulting wave front is indicated with magenta contours for (1) 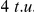 and (2) 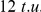 immediately prior the S2 stimulus (indicated by vertical arrow). Lower panel shows state variables excitability 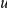 (black) and recovery 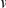 (scaled using 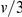) (red) on the cross section indicated by a thick black line in the upper panel. (**B**) ‘Classical vulnerable zone’. Lower panel shows state variables in the refractory tail of the S1 wave (inset from figure A indicated by dotted lines). Upper panel shows regimes of responses to the S2 stimulation as a function of position and amplitude. Lowest S2 amplitudes that result in a connected wave front are indicated as black crosses, stimulations that result in spiral wave dynamics are indicated blue. Stimulations with lower amplitudes (under curve) result in no wave propagation. A 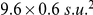 stimulus was applied for 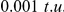 with different amplitudes. System size was 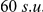. S1 stimulus was applied at 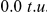, and S2 stimulus was applied at 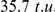

Figure 1 illustrates the setup of the pinwheel experiment and demonstrates the vulnerability of an excitable medium in the dRDM model without deformation (Eqs.(1),(2)). In a pinwheel experiment a ‘secondary stimulus’ (S2) is applied in the back of a previously initiated ‘S1 wave’ (see Figure 1A). The wave initiated by the S2 stimulation can be blocked by the refractory tail (lower panel of Figure 1) towards the propagation direction of the S1 wave and can only propagate in other directions (see schematic successive front positions in upper panel of Figure 1A). As a result a pair of counter rotating spiral waves occurs. The upper panel of Figure 1B illustrates the vulnerable zone of the non-deforming excitable medium. Blue dots symbolize S2 stimuli for which spiral waves were initiated. The black crosses mark the lowest stimulation strengths which result in no conduction blocks and thus to connected wave fronts. Stimulations with positions and strengths under the lower bound of these regimes resulted in no wave initiation (the threshold of excitation). This unidirectional block is caused by a gradient in excitability due to the recovery tail of the S1 wave [22]. The lower panel of Figure 1B shows an inset of the voltage and the recovery variable in the tail of the S1 wave. We see a monotonic decrease of the recovery variable v which causes the monotonic decrease of excitability (compare upper panel of Figure 1B) in the tail of the wave, which explains the block of the S2 wave in the forward direction. We refer to the vulnerable zone, which is formed by the recovery tail of a wave, as the ‘classical vulnerable zone’ in this work. The ‘classical vulnerable zone’ has been shown previously to be an inherent property of excitable media [16].

## Results

### ‘Classical Vulnerable Zone’ in Deforming dRDM System

**Figure 2:**
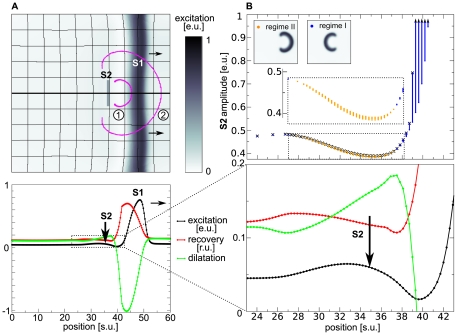
Mechanically induced vulnerability. (**A**) Spiral wave initiation by S2 stimulus in mechanically caused vulnerable zone. Upper panel shows 2D medium. The resulting wave front is indicated with magenta contours for (1) 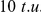 and (2) 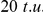 after S2 stimulus was applied. Lower panel shows main variables transmembrane potential 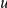 (black), recovery (scaled using 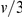) (red) and the dilatation (scaled using 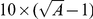) (green) on the cross section indicated by a thick black line in the upper panel, immediately prior the S2 stimulus (indicated by vertical arrow). (**B**) Mechanically induced vulnerability. Lower panel shows state variables in the refractory tail of the S1 wave (inset from figure A indicated by dotted lines). Upper panel shows regimes of responses to the S2 stimulation as a function of position and amplitude. Smallest S2 amplitudes that result in a connected wave front are indicated as black crosses, stimulations that result in classical spiral wave dynamics (block towards S1) are indicated blue (regime *I*). Stimulations that result in the new regime of spiral wave dynamics (block retrogradely to the S1 wave) are indicated as orange points (regime *II*). Stimulations with lower amplitudes (under curve) result in no wave propagation. The inset magnifies stimulations that result in spiral waves in mechanically caused vulnerable zone. A 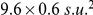 stimulus was applied for 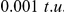 with different amplitudes. System size was 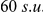 and 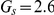. S1 stimulus was applied at 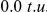 and the S2 stimulus was applied at 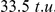.

Applying the pinwheel experiment for the deforming dRDM system (Eqs.(1)-(8)) we find the ‘classical vulnerable zone’ slightly altered. The top panel of Figure 2B illustrates the vulnerable zone for the deforming dRDM system. Following the notation from Figure 1B, the stimulations at positions and amplitudes that result in classical spiral wave initiation are indicated in blue, and the lowest stimulation strengths that cause connected wave fronts are indicated as black crosses. A comparison of the top panels of Figure 2B and Figure 1B shows that the ‘classical vulnerable zone’ (regime *I*) is also present in the deforming system. The ‘classical vulnerable zone’ in the deformed case shows a steeper excitability gradient compared to the undeformed system. As a result, the threshold of excitation for the undeformed system is higher in the ‘classical vulnerable zone’ compared to the deforming system. For instance, at position 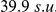 a minimal S2 amplitude of 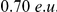 is required in the deformed case, whereas for the undeformed case a minimal S2 amplitude of 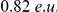 is required. This can be explained by the depolarizing stretch activated current 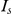, that is present for positions smaller than 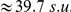, where local stretch develops (lower panel of Figure 2B) and leads to an increase in 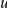 and thus to a decreased threshold of excitation.

### New Mechanism of Spiral Wave Initiation

**Figure 3:**
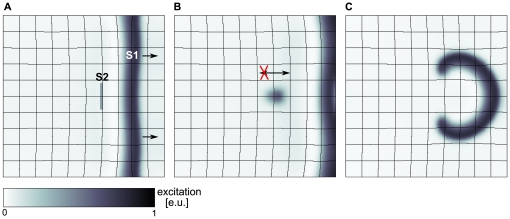
New regime of spiral wave initiation. (**A**) S2 stimulus is applied in the mechanically induced vulnerable zone of S1 wave (arrows indicate propagation direction). (**B**) Unidirectional block of S2 wave retrogradely to propagation direction of S1 wave (indicated by arrows), 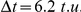 after S2 stimulus. (**C**) Figure eight reentry pattern evolved from wave block of S2 wave response (see B), 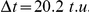 after S2 stimulus. For the S2 stimulus a 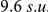 long and 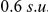 thick electrode of 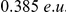 was applied at position 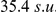 for 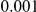 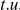. System size was 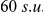. S1 stimulus to initiate the S1 wave was applied at 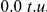, S2 stimulus was applied at 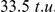. System and simulation protocol as in Figure 2.

In addition to the ‘classical vulnerable zone’ we find a new regime of spiral wave initiation for the deformed case. For stimulations at positions 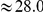 to 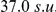, depicted as orange dots in the upper panel of Figure 2B, we see that the propagation of the S2 wave is blocked oppositely to the propagation direction of the S1 wave. As a result, a pair of counter rotating spiral waves is initiated (upper panel Figure 2A) (regime *II*) with rotation directions opposite to spirals initiated by the classical mechanism (upper panel Figure 1A) (regime *I*). This process of mechanically caused conduction block and spiral wave initiation is illustrated in Figure 3. We see an S2 stimulation (Figure 3A) that results in a wave that can propagate away from the S1 wave but fails to propagate towards it (Figure 3B), forming a pair of counter rotating spirals (Figure 3C).

The mechanism of this new regime of spiral wave initiation can be understood from Figure 2B. We observe a non-monotonous dependence of the threshold for wave initiation by an S2 stimulus (lower boundary of curve in top panel of Figure 2B) on the distance from the S1 wave. The medium is most excitable (lowest threshold) at position 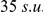, from where the threshold gradually increases with larger distance from the S1 wave to a steady state value. As a result, a stimulation around the maximal excitability in the medium may initiate a wave that is able to propagate towards the S1 wave, but is blocked in the opposite direction.

How does deformation cause this new vulnerability? In the lower panel of Figure 2B we can see the state variables of the dRDM model in the vulnerable zone. The transmembrane potential 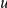 is indicated as a black line, the recovery state as a red line and stretch as a green line. We see, that in the back of the S1 wave where the medium recovers (from 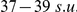), local stretch develops in the medium. The stretch in the medium causes depolarizing stretch activated current 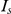, which leads to an increase of 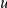. In general, an increase of 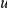 brings the system closer to the threshold value and thus decreases the threshold of the S2 stimulation amplitude, which explains the decrease of the S2 threshold in the ‘classical vulnerable zone’. However, further behind the back of the S1 wave another process takes place. The increased 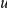 causes the recovery state 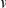 to increase, which in turn decreases the excitability of the medium. In conclusion we can state that the threshold minimum and gradient emerges due to the depolarization of the tissue by stretch activated current 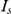 (Eq.(8)). Note, that in general, the decrease of excitability caused by slow depolarisation is a well known phenomenon, called accommodation which has been studied in electrophysiology since 1936 [23],[24]. A previous study [13] also showed that this phenomenon can result in block of waves during spiral wave rotation. Here we show another mechanically induced manifestation of accommodation in RDM systems.

### Dependence of the New Mechanism on Stimulation Parameters

**Figure 4:**
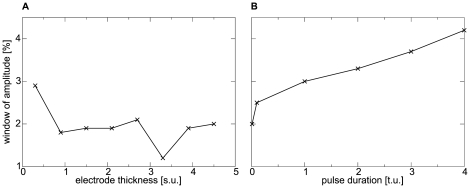
Dependence of the new mechanism on stimulation parameters. (**A**) Window of amplitude against electrode thickness. (**B**) Window of amplitude against pulse duration. System and stimulation protocol as in Figure 2.

We tested how the protocol of stimulation affects the new mechanism of spiral wave initiation. For this, we studied the effect of electrode shape (thickness) and duration of a S2 stimulus, applied in the position of maximal excitability (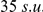), on the window of stimulus strengths that initiate spiral waves. We found that the thickness of the stimulated region does not affect the parameter window significantly (Figure 4A). However, it turned out that a longer stimulation pulse approximately doubles the parameter window to a maximal window size of 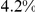 (Figure 4B).

### Mechanically Initiated Spiral Waves

**Figure 5:**
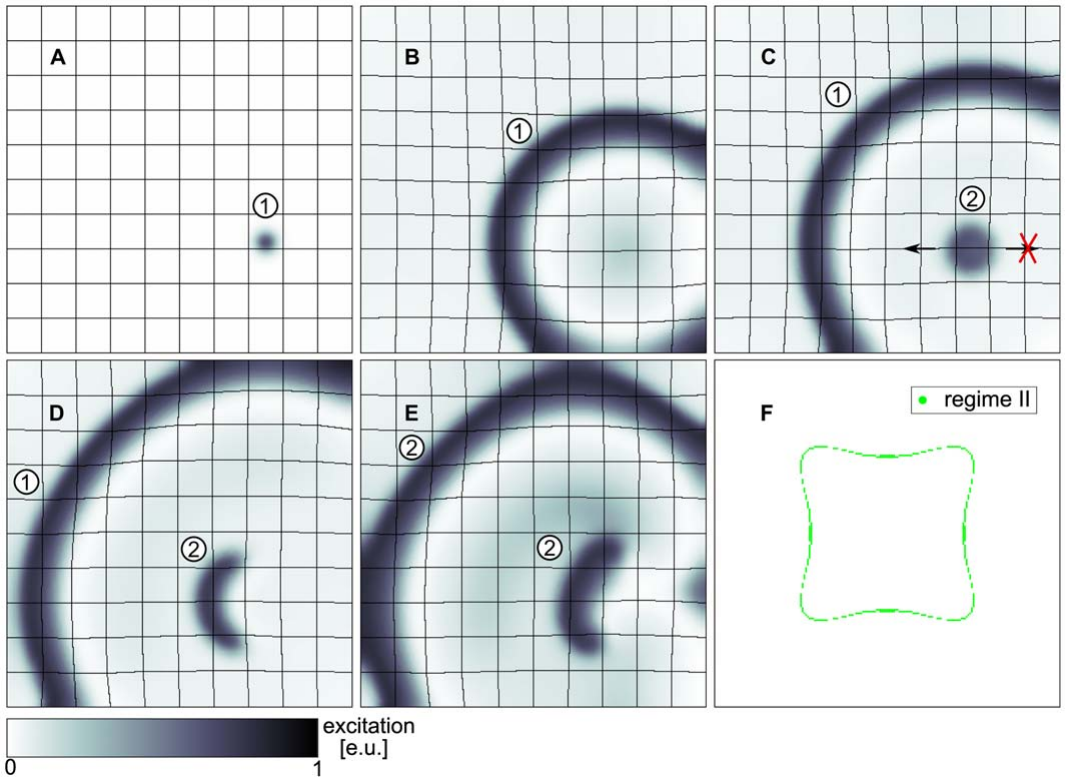
Spiral wave formation following a point stimulus. (**A**) A wave (1) forms as response to a point stimulus (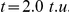). (**B**) Wave (1) propagates radially and causes stretch and initiation of a wave (2) due to depolarizing stretch activated current 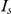 (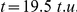). (**C**) The wave (2) is unidirectionally blocked (indicated by arrows) in the mechanically induced vulnerable zone (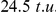). (**D**) Wave (2) forms a pair of counter rotating spiral waves (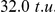). (**E**) Spiral wave pair (2) after one rotation (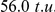). (**F**) Enclosed by green region: pacemaking regime, excluded by green region: quiescence regime, green region: critical region where a point stimulus initiates spiral wave dynamics. System size as in Figure 2 and 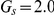.

Although the interval of S2 stimulation strengths, for which spiral wave dynamics is induced, is narrow, it may still be an important mechanism, because the mechanically caused vulnerable zone is located at the area of maximal excitability in the medium. This is illustrated by the following examples. It has been shown previously [12], [14] that a point stimulus in an electromechanical system can produce pacemaking activity. This is because a radially spreading excitation-contraction wave tends to stretch and thus depolarizes the medium by stretch activated current in the vicinity of the initial point stimulus. This stretch activated current can initiate a new wave. The strength of this stretch activated current depends on the degree of stretch of the medium which itself is affected by many other factors, such as boundary conditions and elastic properties of the material. Furthermore, the magnitude in stretch depends on the location of the initial stimulus in the medium: if it is closer to the center the stretch amplitude is maximal, and decreases when the pacemaker position shifts to the boundary of the medium [14]. It turns out that this effect can also initiate spiral waves via the new mechanism reported in this paper. If a point stimulus is applied in the system within the inner region of the medium enclosed by the green region in Figure 5F, then sustained pacemaking activity emerges [14]. However, if the initial stimulus is applied in the region outside the green region in Figure 5F, then the resulting stretch in the vicinity of the initial stimulation site is not sufficient to stimulate an additional pulse. Furthermore, if the initial stimulus is applied in the green area, it leads to a ‘close to threshold’ excitation of the medium and spiral wave generation via the new mechanism. Figures 5A-E illustrate this phenomenon (see also Video S1). We see that wave 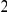 is blocked unidirectionally towards the boundary of the medium (Figure 5D) initiating a pair of counter rotating spiral waves via the new mechanism (compare Figure 3). The surface area of this ‘critical region’ is 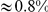 of the total surface area.

**Figure 6 pone-0027264-g006:**
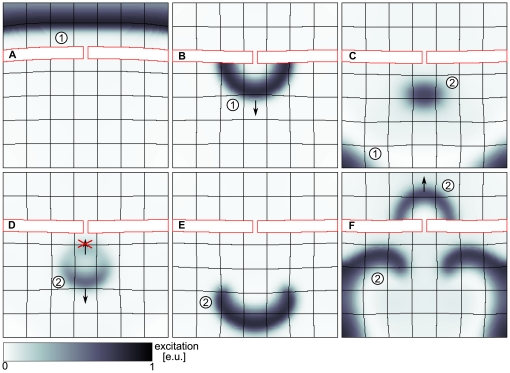
Spiral wave formation following a wave diffraction at an isthmus. (**A**) A plain wave (1) propagating towards an isthmus (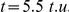). (**B**) Wave (1) diffracted at the isthmus (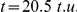). (**C**) Wave (1) produces stretch activated current that initiates wave (2) (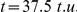). (**D**) Initiated wave (2) is unidirectionally blocked in the mechanically induced vulnerable zone of wave (1) (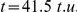). (**E**) Wave (2) forms a counter rotating spiral wave pair (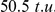). (**F**) Spiral wave pair after 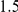 rotations (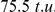). System size 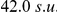 and 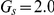. Stiffness of the isthmus (contoured red) is twofold the stiffness in medium (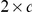).

Such a scenario of mechanically induced spiral wave formation can also occur without a point stimulus. In [14] it has been shown that the curvature of a wave in a dRDM system similar to that used in this paper causes an asymmetric strain distribution in the medium. Curvature effects have been shown to be important for spiral wave initiation [25], [26]. One physiological example how a curvature of a wave front can be created is the diffraction of a travelling wave at an isthmus, which was studied extensively in cardiac electrophysiology [27]. In Figure 6 we show that such a diffraction event can also result in spiral waves initiation via the new mechanism. We see that the initial wave 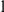 forms a curved wave front after diffraction at an isthmus. The curvature of wave 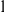 leads to a region of maximal stretch in the focus of the curved wave 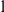 which initiates an additional wave 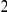. Wave 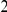 is blocked retrogradely to the propagation direction of wave 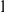 forming two spirals via the new mechanism (see also Video S2).

**Figure 7:**
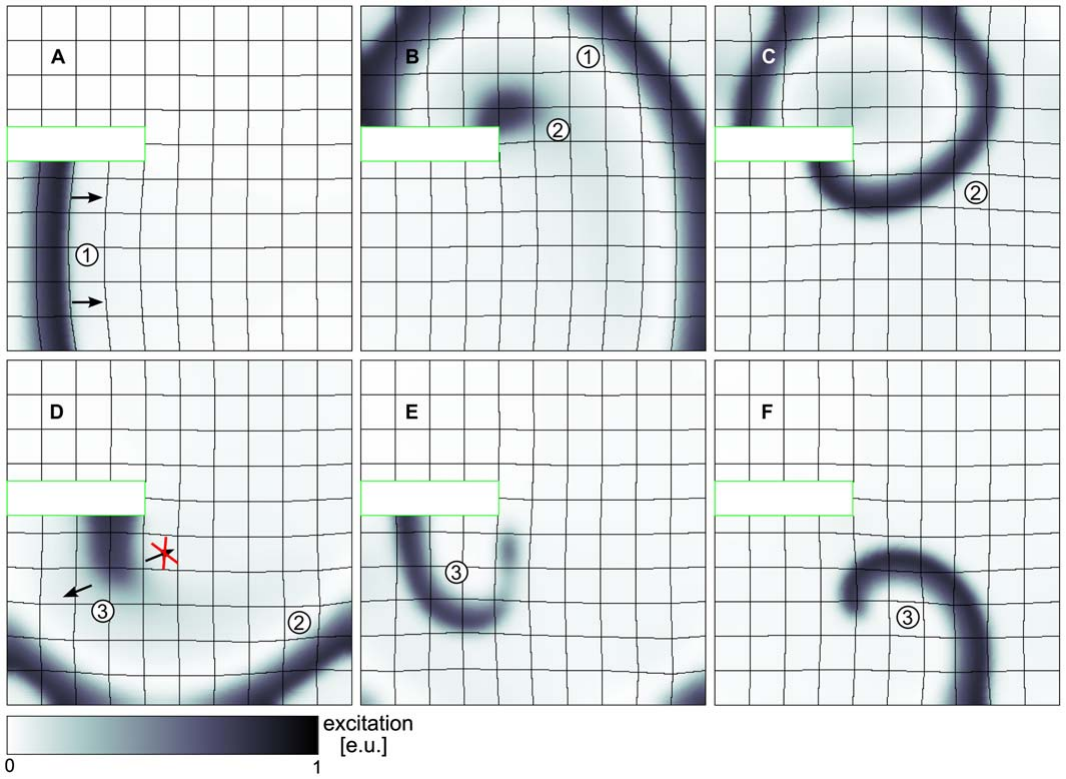
Spiral wave formation following a wave deflection. (**A**) A plain wave (1) propagating around a non-conducting static block (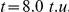). (**B**) Stretch caused by wave (1) initiates new wave (2) (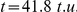). (**C**) Wave (2) is propagating around non-conducting static block (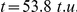). (**D**) Stretch caused by wave (2) initiates wave (3) in mechanically induced vulnerable zone of wave (2) causing unidirectional block of wave (3) (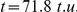). (**E**) Wave (3) forms spiral wave (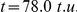). (**F**) Spiral wave (3) after one rotation (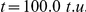). System size 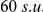 and 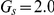. The static block is contoured green.

Another more complex scenario for mechanical spiral wave initiation is presented in Figure 7 (see also Video S3). We see a wave front propagating around an obstacle, where it gets curved and leads to a new wave initiation (Figure 7A,B). This new wave 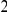 itself is curved and initiates another wave 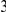 (Figures 7C,D). However, this new wave 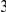 is unidirectionally blocked retrogradely towards the propagation direction of wave 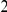, and thus a rotating spiral wave is initiated.

Note, that in all examples shown above the formation of new spiral waves is caused by mechanically induced ‘close-to-threshold’ stimuli in the area of maximal excitability, and thus according to the mechanism of spiral wave initiation reported in this article.

## Discussion

In this paper we show that deformation can substantially affect the vulnerability of an excitable medium. At longer coupling intervals, deformation induces a new vulnerable zone different from the classical vulnerable zone in the dRDM model. In this mechanically induced vulnerable zone we found a new mechanism of spiral wave initiation which results in spiral wave pairs that rotate counter to the directions of spirals resulting from the classical vulnerable zone. The mechanically induced vulnerable zone is located at a region of maximal excitability in the back of an excitation wave. As a result, this mechanism of spiral wave initiation is relevant for weak stimuli, which can for example be caused by stretch activated currents. Using diffraction at an isthmus and a deflection on a non-conducting inhomogeneous medium as examples, we show that when curvature is introduced to a wave of excitation, it can lead to asymmetric stretch distributions causing weak stimuli to initiate spiral waves by the mechanism shown in this article.

All studies were performed with the dRDM model, which describes the mechanics of the medium in a discrete formulation. Another approach of modeling electromechanics uses continuous modeling frameworks. However, the dRDM approach has been shown to reproduce RDM phenomena previously found with a continuous modeling framework [14]. Thus we expect our results to hold true for continuous descriptions of cardiac mechanics.

The mechanically caused vulnerable zone is located at longer coupling intervals compared to the ‘classical vulnerable zone’. It would be interesting to see if this new vulnerability can be seen in an experiment for electrical stimulation of cardiac tissue or mechanical stimulation mimicking the onset of the deadly heart arrhythmia ‘commotio cordis’, a state of chaotic excitation patterns following an impact on the heart tissue.

Note, that in our simulations the parameter range for the initiation of spiral waves, and for the generation of new pulses via stretch are close to each other. In [28] it has been suggested, that stretch can induce new pulses in cardiac tissue. We believe therefore, that under these circumstances it will also be possible to induce spiral waves by the mechanism shown in Figures 5–7 in this paper.

In this paper isometric boundary conditions (fixed boundaries) were applied. The change of the boundary conditions affects the stretch magnitude and distribution in the medium, and accordingly may shift parametric ranges of the observed phenomena. However, changes in boundary conditions do not change the basic effects of stretch activated currents, and thus conditions for the emergence of the mechanically caused vulnerability. Therefore, we expect that with other mechanical boundary conditions we will get similar results, provided that sufficiently large stretch is developed in the medium. Quantifying the effects of boundary conditions and geometry may be the subject of a following study.

We performed this study in a phenomenological low dimensional model of cardiac excitation. However, we think that the results will be reproduced in more detailed models, because the mechanism is based on the accommodation effect, which can be perfectly reproduced by ionic models.

The phenomenon ‘superexcitability’ has been reported in various experimental studies [29], [30], and may be caused by factors not related to mechano-electrical feedback. As the basic requirement for our mechanism of spiral wave initiation, besides a threshold stimulus, is only superexcitability, we can expect that it may also work in situations when it is not mechanically induced.

## Supporting Information

Video S1 Spiral wave formation following a point stimulus. Compare Figure 5.
(MPG)

Video S2 Spiral wave formation following a wave diffraction at an isthmus. Compare Figure 6.
(MPG)

Video S3 Spiral wave formation following a wave deflection. Compare Figure 7.
(MPG)
